# Biotechnology and Biomaterial-Based Therapeutic Strategies for Age-Related Macular Degeneration. Part I: Biomaterials-Based Drug Delivery Devices

**DOI:** 10.3389/fbioe.2020.549089

**Published:** 2020-11-03

**Authors:** Nahla Jemni-Damer, Atocha Guedan-Duran, María Fuentes-Andion, Nora Serrano-Bengoechea, Nuria Alfageme-Lopez, Felix Armada-Maresca, Gustavo V. Guinea, José Pérez-Rigueiro, Francisco Rojo, Daniel Gonzalez-Nieto, David L. Kaplan, Fivos Panetsos

**Affiliations:** ^1^Neuro-Computing and Neuro-Robotics Research Group, Complutense University of Madrid, Madrid, Spain; ^2^Innovation Group, Institute for Health Research San Carlos Clinical Hospital (IdISSC), Madrid, Spain; ^3^Department of Biomedical Engineering, Tufts University, Medford, MA, United States; ^4^Silk Biomed SL, Madrid, Spain; ^5^Ophthalmology Service, La Paz University Hospital, Madrid, Spain; ^6^Center for Biomedical Technology, Universidad Politécnica de Madrid, Madrid, Spain; ^7^Department of Material Science, Civil Engineering Superior School, Universidad Politécnica de Madrid, Madrid, Spain; ^8^Biomedical Research Networking Center in Bioengineering, Biomaterials and Nanomedicine (CIBER-BBN), Madrid, Spain

**Keywords:** retinal pigment epithelium, Bruch’s membrane, retina, biomaterials, neuroprotection, ocular drug delivery, nanocarriers, VEGF

## Abstract

Age-related Macular Degeneration (AMD) is an up-to-date untreatable chronic neurodegenerative eye disease of multifactorial origin, and the main causes of blindness in over 65 years old people. It is characterized by a slow progression and the presence of a multitude of factors, highlighting those related to diet, genetic heritage and environmental conditions, present throughout each of the stages of the illness. Current therapeutic approaches, mainly consisting of intraocular drug delivery, are only used for symptoms relief and/or to decelerate the progression of the disease. Furthermore, they are overly simplistic and ignore the complexity of the disease and the enormous differences in the symptomatology between patients. Due to the wide impact of the AMD and the up-to-date absence of clinical solutions, the development of biomaterials-based approaches for a personalized and controlled delivery of therapeutic drugs and biomolecules represents the main challenge for the defeat of this neurodegenerative disease. Here we present a critical review of the available and under development AMD therapeutic approaches, from a biomaterials and biotechnological point of view. We highlight benefits and limitations and we forecast forthcoming alternatives based on novel biomaterials and biotechnology methods. In the first part we expose the physiological and clinical aspects of the disease, focusing on the multiple factors that give origin to the disorder and highlighting the contribution of these factors to the triggering of each step of the disease. Then we analyze available and under development biomaterials-based drug-delivery devices (DDD), taking into account the anatomical and functional characteristics of the healthy and ill retinal tissue.

## Introduction

Age-related macular degeneration is a chronic neurodegenerative eye disease of multifactorial origin, characterized by the appearance of alterations in the central part of the retina, the macula. According to the WHO, there are 196 million cases worldwide and are expected to reach 288 million by 2040, making AMD one of the leading causes of irreversible loss of vision in people over 65 years in developed countries (Evans et al., 2004; Ferris et al., 2005; Lim et al., 2012; Garciìa and Martinez, 2013; IAPB Vision Atlas, 2017; World Health Organization, 2018). In the United States there are more than 2 million people affected by AMD and it is estimated that they will exceed 4 million by 2030 (Friedman et al., 2004; Klein et al., 2011; Friedman et al., 2012; Degeneration et al., 2017). In Western Europe, the IAPB estimates 4.8 million cases of AMD for 2020, making it the second most common cause of eye disease, preceded only by cataracts (Damián et al., 2006; Casado, 2009; Klein et al., 2011; Degeneration et al., 2017; Jeffries et al., 2017).

Despite the great research efforts dedicated in the development of AMD therapies, nowadays available treatments only are able to treat symptomatology and/or slowing down the progression of the disease.

In the present paper we briefly analyze the biomolecular and cellular processes responsible for the development of the AMD, and we review available and under development biomaterials-based drug-delivery devices (DDD) for possible treatments of disease.

## Natural History

### Risk Factors

The pathogenesis mechanisms of AMD are heterogeneous and not yet understood (Lacour et al., 2002; Ambati and Fowler, 2012; Kabiesz and Nowak, 2015; Michalska-Małecka et al., 2015), however, it is known that one of the main risk factor for AMD is aging (Lacour et al., 2002; Ardeljan and Chan, 2013; Daneault et al., 2016). Other risk factors are sex (higher rates in women), race (higher rates in the Caucasian population) and environmental factors that increase oxidative stress in the RPE, such as: blue light exposure, smoking, alcohol consumption, obesity, low antioxidant diet (e.g., lack of vitamins A and E, zinc, lutein and omega-3 fatty acids) as well as systemic factors associated with cardiovascular risks (Thornton et al., 2005; Michalska-Małecka et al., 2015). The risk of AMD is also increased by ocular factors such as macular pigment optical density, iris pigmentation (less risk in dark eyes), cataract surgery and high refractive errors (Michalska-Małecka et al., 2015). In addition, several genetic factors have been reported (Table 1).

**TABLE 1:** List of possible genes that have a determining influence on AMD (Klein et al., 2005; Rivera et al., 2005; Zareparsi et al., 2005; Khan et al., 2006; Nozaki et al., 2006; Ratnapriya and Chew, 2013; Michalska-Małecka et al., 2015; Sergejeva et al., 2016).

Polymorphisms of the complement factor H	CFH
Age-related maculopathy susceptibility 2	ARMS2/LOC 387715
Genes of the complement pathway	C2, CFB, C3 and CFI
Genes encoding inflammatory factors	C-X-C motif chemokine receptor 1 - CXCR1-, toll-like receptor 3 - TLR3-, TLR4, human leukocyte antigen -HLA-
Cholesterol/lipid metabolism	Lipase C - LIPC-, cholesteryl ester transfer protein - CETP-, ATP-binding cassette A-subclass - ABCA1-, ABCA4, apolipoprotein E -APOE-
Collagen	COL10A1 and COL8A1
Extracellular matrix	Cystatin C - CST3-, matrix metalloproteinase -MMP- 9-, tissue inhibitor of metalloproteinases-3 - TIMP3-, fibulin
Angiogenesis regulation-involved factors	Vascular endothelial growth factor A -VEGFA-

### Clinical Aspects

**FIGURE 1:**
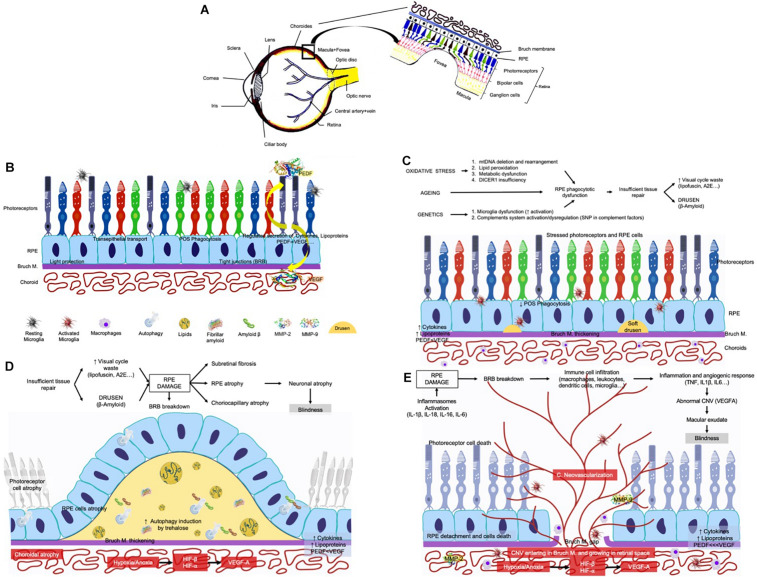
Schematic representation of the eye and the stages of the AMD. **(A)** The eye and the retina. **(B)** Normal retina in a cross section of the eye where appear: the layer of the photoreceptors, the choroid, the RPE and the Bruch’s membrane (BrM). The main functions of the normal RPE are: (1) transport of nutrients, ions, and water (2) absorption of light and protection against photooxidation, (3) re-isomerization of all-*trans*-retinal into 11-*cis*-retinal, which is a key element of the visual cycle, (4) phagocytosis of shed photoreceptor membranes, and (5) secretion of various essential factors for the structural integrity of the retina. POS, photoreceptors outer segment; BRB, blood–retinal barrier; PEDF, pigment epithelium-derived factor, the most potent inhibitor of angiogenesis in the mammalian eye; VEGF, vascular endothelial growth factor. VEGF is essential for development and maintenance of functionally efficient retinal vasculature as well as for integrity of the RPE, BrM, and choroidal endothelial cells. **(C)** Early and intermediate AMD phases in a cross section of the eye. Without apparent cause, debris from the RPE cells layer as well as from the surrounding tissues begin to accumulate between RPE and BrM forming clusters called drusen. **(D)** Progress of the dry AMD in a cross section of the eye where the clusters called drusen start growing in size and accumulate as the disease progresses. The accumulation of drusen and the degeneration of RPE provokes the beginning of photoreceptor’s atrophy. **(E)** The progress of wet AMD. As drusen accumulate, they can cause inflammation. Inflammatory cells are then recruited by the retina, and these cells, together with the RPE begin to release vascular growth factors (mainly VEGF) that cause growth of the blood vessels. In this case, the degeneration is characterized by choroidal neovascularization, neovessels penetrating the BrM, presence of inflammatory cells, exudates of lipid content, hemorrhage, and the destruction of the RPE and photoreceptors (wet AMD).

**FIGURE 2:**
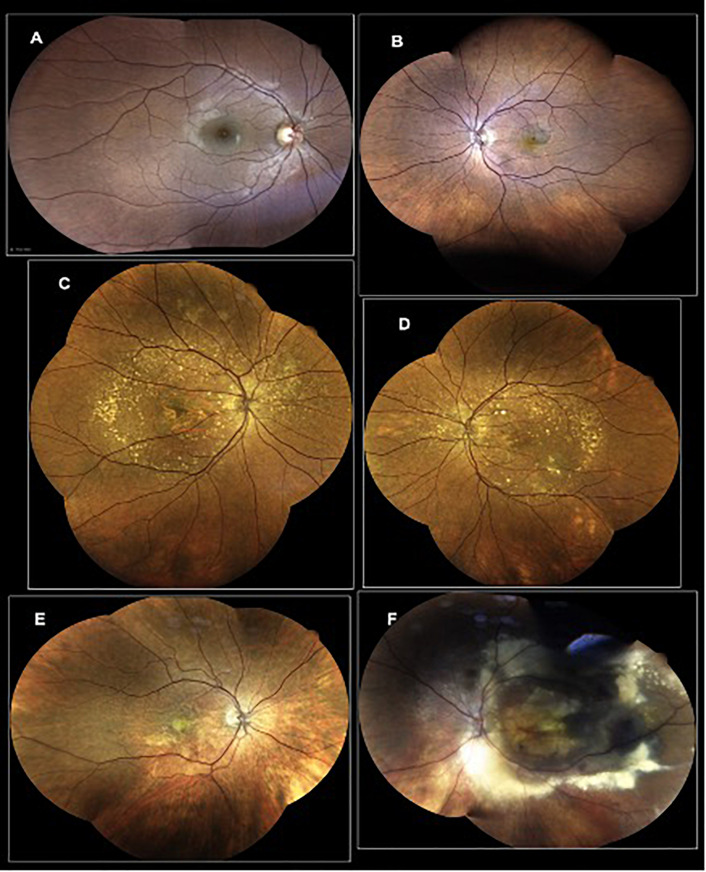
Representative photographs of AMD-affected eyes. **(A)** Eye of a healthy subject; **(B)** AMD patient with macular hemorrhage and active exudation; **(C)** AMD patient with wide macular geographic atrophy and several druses in the back of the eye; **(D)** AMD patient with macular pigment changes, several druses as well as a macular hemorrhage secondary to an active neovascular complex; **(E)** patient with an active macular neovascular complex, secondary to an AMD; **(F)** AMD patient with an active AMD with a pseudo-tumoral lesion.

An outline of the stages involved in the disease, and the fundus images of affected patients are shown in Figures 1, 2, respectively. The early stages of AMD are detected by the presence of drusen between the basal membrane of the RPE and the Bruch’s membrane (BrM). Drusen are lipids, cellular and metabolic debris that originate in the RPE and surrounding tissues. In more advanced stages of the disease, these deposits may be also found between the RPE and the neuroretina. In both cases, metabolic abnormalities in the RPE cells is one of the causes of the formation of drusen (Winkler et al., 1999; Anderson et al., 2002; McConnell and Silvestri, 2005; Damián et al., 2006; Nowak, 2006; Jager et al., 2008). Depending on the number and extension of the drusen and the degree of the macular atrophy, AMD is customarily classified into three stages (Table 2): Early AMD; characterized by the presence of a few, small or medium-sized (<124 μm) drusen and minimal RPE cell alterations leading to some pigmentary anomalies (hypo- and hyper-pigmentation). Patients are normally asymptomatic. Intermediate AMD; characterized by the presence of one or more large (>124 μm) drusen, together with geographic atrophy that does not reach the central area of the macula. Patients are either asymptomatic or complain of decreased contrast sensitivity, abnormal scotopic vision or blurred vision while reading. Advanced AMD; either manifested in dry (D-AMD) or wet (W-AMD) form. D-AMD is characterized by the presence of large white or yellow drusen, the RPE atrophy and the photoreceptors’ deterioration. Patients suffer from a decreased visual acuity and metamorphopsias. W-AMD is characterized by choroidal neovascularization, macular edema, aneurysms, hemorrhages, RPE and/or retinal detachment and photoreceptors death. Patients present important loss of visual acuity or total blindness, metamorphopsias and photopsia.

**TABLE 2:** AMD stages: pathological characterization and clinical aspects.

Type of AMD	“Early” AMD	“Intermediate” AMD	“Advanced” AMD	
	No maculopathy	Dry/atrophic	Dry/atrophic	Wet/exudative
Incidence	–	–	80% cases	20% cases
Progression	Slow progression (lasting years)	Slow progression (lasting years)	Slow progression (lasting years)	Fast progression (lasting a few months)
Physiological changes/Clinical aspects	Small drusen (<124 μm). Minimal pigmentary anomalies.	One or more large drusen (>124 μm). Geographic atrophy not reaching the central area of the macula.	Large white or yellow drusen, cellular deterioration. Final Stage: Geographic atrophy, BrM thinning and disruption.	Choroidal neovascularization, Macular edema, aneurisms, hemorrhages, RPE and/or retinal detachment, photoreceptors death. Final Stage: Disciform scar, BrM thinning and disruption.
Impact on visual acuity	Normally asymptomatic	Normally asymptomatic, abnormal scotopic vision or blurred vision while reading	Decreased visual acuity and metamorphopsias	Important loss of visual acuity or total blindness, metamorphopsies and photopsia
Palliative Treatment	Regular medical observation for early detection	Antioxidant vitamin + zinc and mineral supplement	Antioxidant vitamin + zinc + mineral supplement. Complement factors’ inhibitors (C3, C5). Visual cycle modulators (Fenretinide)	Pharmacological treatment (Anti-VEGF intravitreal injection). PDT – Photo-Dynamic Therapy with Verteporfin, Surgery

*RPE, retinal pigment epithelium; CPZ, choriocapillary, pigmented zones; BrM, Bruch’s membrane; VEGF, vascular endothelial growth factor.*

### Pathogenesis

Most pathways and causes of the AMD are very well-established. However, this fact does not imply a straightforward identification of the AMD-triggering mechanisms. Such identification is challenging because of the multifactorial and extremely complex etiology of this disease: (I) It is not still clear if all of them have been identified, (II) None of them has been proved as the sole cause of the disease, and (III) Synergistic relations between causes that determine each phase triggering of the disease has not yet been identified. As we have already mentioned, AMD is the result of several molecular and cellular processes of different origins that interact with each other. Oxidative stress and inflammation are at the origin of neovascularization processes. Neovascularization, in turn, affects the function of the retina and causes additional inflammation, establishing a vicious circle that encourages the progress of AMD.

#### Oxidative Stress

Oxidative stress refers to an imbalance between the number of oxidants (reactive oxygen species) produced in a cell and the capacity of different scavenging enzymes and other molecules with anti-oxidant properties to reduce such an imbalance (Winkler et al., 1999; Beatty et al., 2000; Lin and Beal, 2006; Ambati and Fowler, 2012; Jarrett and Boulton, 2012; Cuenca et al., 2014; Hanus et al., 2015; Tang and Le, 2016). The antioxidant effect is achieved by a cell defense reducing process, carried out by superoxide dismutase, peroxidase, and catalase enzymes.

At early and intermediate stages (I, II) of the disease, key factors for the development of AMD are increased levels of oxidative stress and toxin accumulation in the retina in connection with dysfunction and atrophy of RPE cells responsible for the elimination of toxic accumulations (Winkler et al., 1999; Ambati and Fowler, 2012; Jarrett and Boulton, 2012). Toxic accumulations in the retina can form intracellular remains (lipofuscin and oxidized mitochondria), extracellular waste (drusen) or reactive oxygen species, all of them being some of the multifactorial causes of AMD (Ambati and Fowler, 2012). Among the different waste products being accumulating as the retina ages and as the disease progresses, we would like to underlie the accumulations of lipofuscin -a mixture of oxidized proteins, lipids, carbohydrates, and other types of bioorganic molecules- in the RPE, similar to the accumulation of β-amyloid and tau-protein in other neurodegenerative diseases. The accumulation of these molecules seems to be related to a possible vulnerability of RPE cells to oxidative stress (Crouch et al., 2015; Handa et al., 2019).

Lipids accumulation is considered one of the causes of the inflammation process as well as of the accumulation of complement factors which occurs later. Particularly important is the cholesterol because of the lack of recycling and disposal processes of this type of molecule (Handa et al., 2019). Another component present in lipofuscin is A2E, a natural product of the visual cycle, which accumulates as the disease progresses (Crouch et al., 2015). Closely related to smoking habits, cadmium is another AMD-triggering accumulated molecule. It is one of the toxic products present in tobacco, and it is accumulated in the retinas during the evolution of the disease (Wills et al., 2008).

#### Altered Cell Functions

Atrophy and disfunction of RPE cells appear in the intermediate phase. These cells are responsible for the elimination of toxic accumulations in the retina, so their atrophy provokes uncontrolled growth of such cumuli which, in turn, generates positive feedback to the previous mechanism. In the dry stage, toxin accumulation also appears within the cells of the RPE (Ambati and Fowler, 2012; Kinnunen et al., 2012; Tarallo et al., 2012).

Also, local inflammation and functional deficits of retinal glial cells impede the development of the molecular processes that are responsible for retinal tissue repair (Cuenca et al., 2014; Tang and Le, 2016). Glia has a strong contribution to AMD development. Under physiological conditions microglia and macroglia (astrocytes and Müller cells) are in continuous communication, reporting on neural activity and maintaining immunomodulatory activity that is responsible for neuroprotection and regulation of cellular activity. In this way, the activation of microglial cells together with their immune response to the lesions contribute to the development and progression of the disease, provoking tissue degeneration, early changes in the pigmentation of the RPE and the formation of drusen (Polazzi and Monti, 2010; Marín-Teva et al., 2011; Cherry et al., 2014; Tang and Le, 2016; Vilhardt et al., 2017; Rathnasamy et al., 2019).

Finally, molecules damaged by oxidants and cellular deposits due to non-immune damage, induce macrophage infiltration and activation and deposition of complement fragments.

#### Neovascularization

In 10–20% of D-AMD cases, the pathology progresses and the subretinal space is invaded by blood vessels of choroidal origin (choroidal neovascularization) that break into the retinal tissue, provoke inflammation and macular edema, induce serum and/or hemorrhagic detachment of both, the RPE or the neural retina, and, eventually result in fibrovascular disciform scar-associated death of the photoreceptors (Jager et al., 2008; Ambati and Fowler, 2012). Neovascularization alters the geometry, the morphology and the structure of the retina at the macular level that leads to rapid and permanent loss of central vision. Such progression is called wet or exudative AMD (W-AMD). In the transition from D-AMD to W-AMD, the key factor is neovascularization of the retina (Ambati and Fowler, 2012; Cuenca et al., 2014; Tang and Le, 2016). Oxidative stress causes an increase of inflammatory and pro-angiogenic molecules in the subretinal space, which provokes rapid and uncontrolled angiogenesis in the choroid and an invasion of the subretinal space by neovascular vessels.

Chronic inflammation has very important consequences in disease progression. This process triggers the secretion of pro-angiogenic factors by the RPE and other immune cells and also gives rise to further increases in oxidative stress. This increases due to the imbalance between generation and elimination of reactive-oxygen species that creates a toxic retinal microenvironment which eventually leads to the death of the pigment epithelium retinal cells through cell death mechanisms. Furthermore, due to their atrophy, retinal epithelium cells are no longer capable of activating/regulating certain biomolecules derived from the inflammatory process, such as complement factors C3a and C5a (chemotactic agents that cause an increase of leukocytes in the choroid) or some cytokines such as IL1 (Nozaki et al., 2006).

Neovascularization is triggered by increased expression of the vascular endothelial growth factor A (VEGF-A), in charge of increased vascular permeability and vascular endothelial cells recruitment, proliferation and migration. The increased number of macrophages attracted by lipofuscin, cause the secretion of proteolytic enzymes, such as collagenase and elastase (MMP2 and MMP9), which erode BrM, facilitating the penetration of the choroidal neovessels (Risau, 1997; Grossniklaus et al., 2002; Spaide, 2017). In addition, both, astrocytes and Müller cells participate in the development of neo-vessels, releasing angiogenic factors in response to pathogenic stimuli. Müller cells alter the blood-retina barrier, causing infiltration of several blood components including growth factors, cytokines, inflammatory factors and blood-derived immune cells; allowing the growing neo-vessels from the choroid to access the retina through the damaged Bruch’s membrane and the RPE (Bringmann et al., 2009; Nash et al., 2011; Rathnasamy et al., 2019).

Age-related macular degeneration progression is often very slow in the early stages and accelerates in the wet phase. D-AMD usually takes about 5 years to evolve to the wet form (Nussenblatt and Ferris, 2007; Ardeljan and Chan, 2013; Fleckenstein et al., 2018). Although only 10–20% of patients develop W-AMD, this stage is the most important one because it is the origin of more than 80% of severe and very severe vision loss: approximately 40% of patients not correctly treated develop almost complete blindness.

#### Anti-VEGF Drugs

Even though there is no cure for neither form of AMD, there are several EMA/Food and Drug Administration (FDA)-approved therapeutic approaches focusing on symptomatology relief and on slowing down the progression of the disease, all of them exclusively dedicated to the “wet” form of the disease.

Among them, there are several drugs target directly or indirectly the vascular endothelial growth factor (VEGF). These drugs include pegaptanib, ranibizumab, aflibercept, and brolucizumab. Bevacizumab is another drug, non-FDA-approved for W-AMD treating, but it has been used off-label since 2005, with very good results (Martin et al., 2012). These drugs are IVT administered with monthly or bimonthly frequency (Freund et al., 2013) (anti-VEGF agents are summarized in Table 3). Frequent injections are necessary due to the limited volume of drugs that can be injected in the vitreous each time (Tabandeh et al., 2014) and their limited time of efficacy. The risk of bleeding, retinal detachment, endophthalmitis, cataracts, and infection increases with the number of IVT injections (Jager et al., 2004; Falavarjani and Nguyen, 2013).

**TABLE 3:** Drugs for AMD therapy.

Drug	Pegaptanib (Macugen)	Ranibizumab (Lucentis)	Bevacizumab (Avastin)	Aflibercept (Eylea)	Abicipar pegol (Allergan)	Brolucizumab (Novartis)
Characteristics	PEGylated synthetic RNA-based aptamer	Fragment of monoclonal humanized IgG1 antibody	Full monoclonal humanized IgG1 antibody	Recombinant fusion protein (VEGFR-1/2 fused with Fc portion of human IgG)	DARPin: Anti-VEGF designed ankyrin repeat protein	Humanized single-chain (scFv) antibody fragment
Inhibits	VEGF-A_165_ isoform	All VEGF-A isoforms	All VEGF-A isoforms	All VEGF-A isoforms, VEFG-B and PIGF	All VEGF-A isoforms	All VEGF-A isoforms
MW	50 kD	48 kD	149 kD	115 kD	∼34 kD	26 kD
Half-life	8–14 days	6–10 days	4–5 days	7 days	>15 days	2–3 days
Doses	0.3 mg/dose every 2 months	0.5 mg/dose per month	1.25 mg/dose per month	2 mg/dose per month	2 mg/dose every 8–12 weeks	6 mg/dose every 8–12 weeks
Administration	Intravitreal	Intravitreal	Intravitreal	Intravitreal	Intravitreal	Intravitreal
Employment	Used	Used	Used	Used	TRL Phase III	TRL Phase III
FDA	2004	2006	2004*	2011	Declined	2019
EMA	2006	2007	2005*	2012	–	2020
Advantages	Suitable for all AMD types	Higher tissue penetration and affinity than Pegaptanib and Bevacizumab, more efficient than Pegaptanib	Low price. More efficient than Pegaptanib	Efficient up to 2.5 months after injection, Higher affinity than Ranibizumab, Efficient (inhibits more isoforms)	Smaller MW than Ranibizumab, Higher durability and affinity Ranibizumab, High specificity	Smaller MW than Ranibizumab, Higher durability and tissue penetration than Aflibercept and Ranibizumab, High specificity, Rapid systemic clearance
Disadvantages	Only inhibits one VEGF-A isoform Patients lose visual acuity	Fast pharmacokinetics. Only W-AMD. Expensive.	High MW, difficulties in penetrating the deepest layers of the eye, not approved for AMD, only for W-AMD	Only W-AMD, Expensive	Only W-AMD	Only W-AMD
Cost/dose (approx.)	Not anymore used	$1950 (United States) €700 (EU)	$50 (United States) €70 (EU)	$1850 (United States) €600 (EU)	–	$1850 (United States)

IgG, immunoglobulin; VEGF, vascular endothelial growth factor; VEGFR, VEGF receptor; Fc, fragment crystallizable region; DARPin, designed ankyrin repeat proteins; scFv, single-chain variable fragment; PIGF, phosphatidylinositol-glycan biosynthesis class F protein; FDA, US Food and Drug Administration Agency; EMA, European Medicines Agency; TRL, clinical trials. *Approved for metastatic colorectal cancer. Used off-label for W-AMD since 2005.

An intent to overcome this problem was the development of new molecular drugs with different administration routes, for example oral or topic, which are currently under test (McLaughlin et al., 2013; Jackson et al., 2017; Adams et al., 2018). Unfortunately, even though these routes are non-invasive (e.g., ocular drops), drug arrival to the targeted area, the posterior segment of the eye, is hindered by the several barriers the drugs have to cross (Patel et al., 2013), which severely limits drug bioavailability.

For this reason, work is currently underway to extend the IVT administration beyond 2 months by developing DDD (known also as drug delivery systems, or DDS) that allow a controlled release of the drug, widening its action window and thus lowering the number of interventions needed for AMD treatment per year. The ideal DDD should maintain effective levels of the drug for extended periods, reducing the necessary interventions.

Innovative DDD based on biomaterials in the form of hydrogels, liposomes, colloidal particles, nanoparticles, micelles, dendrimers, or combinations of either of them is currently being tested for the delivery of anti-VEGFs drugs to the posterior segment of the eye. Researchers are working with both, synthetic and natural materials, include hyaluronic acid (Awwad et al., 2019), dextran (Yu et al., 2019), Silk fibroin (Lovett et al., 2015), poly (lactic-co-glycolic acid) (PLGA) (Varshochian et al., 2015), poly(ethylene glycol) (PEG) (Yu et al., 2014), *N*-isopropyl acrylamide (NIPAAm) (Liu et al., 2019b), etc. Different materials have been modified or combined to create DDD sustainably releasing the drug through time, either right after injection, or on-demand, responding to precise external stimuli, such as light (Basuki et al., 2017; Jiang et al., 2019).

Age-related macular degeneration represents both a challenge and an opportunity for biologists, physicians and biomedical engineers, among others, since many of the processes that give rise to the disease could be controlled by new therapies employing advanced biomaterials-based carriers loaded with biomolecules or drugs that can be introduced into the eye. In the present paper we review the different biomaterial-based anti-angiogenic delivery systems developed and/or tested as AMD therapeutic strategies. We examine hydrogels, colloidal particles, nanoparticles, and implantable drug-delivery devices, with special attention to their capacity of preservation of drugs bioactivity within the biomaterial.

## Technologies for Controlled Intraocular Drug-Delivery

As any other ocular disease treatment, AMD pharmacological therapy depends on the pharmacodynamics of the involved agents (molecular characteristics of the drug) which are conditioned by the specific tissue barriers (tear film barrier, corneal and conjunctival barrier, blood-aqueous barrier and finally the blood–retinal barrier-BRB), which in term determine the optimal administration route. Blood–retinal barrier (BRB) increase eye’s resistance to exposure to foreign substances but also to pharmacological agents (Cunha-Vaz et al., 2011; Campbell and Humphries, 2013). There is little convection of molecules, since BRB has no cellular components and is selectively permeable to the most lipophilic molecules (Yasukawa et al., 2004; Kang-Mieler et al., 2020). To avoid BRB, medication to the anterior segment of the eye can be delivered topically and by sub-conjunctival or intracameral injections. In the case of the posterior segment, medication can be delivered topically, systemically, periocularly through the suprachoroidal space, and by IVT or subretinal injections (Kang-Mieler et al., 2014, 2020). As mentioned above, AMD treatments often require repeated IVT injections, whose frequency and duration depend on the course of the pathology in the individual patients, and the same occurs in all other eye degenerative diseases. Although certain benefits are obtained, the excessive repetition of the treatment leads to a worsening of the problem. To reduce that repeat burden we need minimally invasive pharma delivery systems, capable to maintain the necessary drug levels throughout several months or even years and this is precisely one of the actual research lines (Kang-Mieler et al., 2020).

Anti-VEGF molecules are proteins with fragile tertiary and quaternary structure, and they are very sensitive to environmental factors, like heat, pH changes, and proteolytic enzymes, so they require strong preservation measures along the administration route, to maintain intact their physical structure and, consequently, their pharmacological activity (Oo and Kalbag, 2016). Furthermore, since they cannot penetrate the structural barriers of the eye, they have to be IVT injected every 1–3 months (Edington et al., 2017).

**FIGURE 3:**
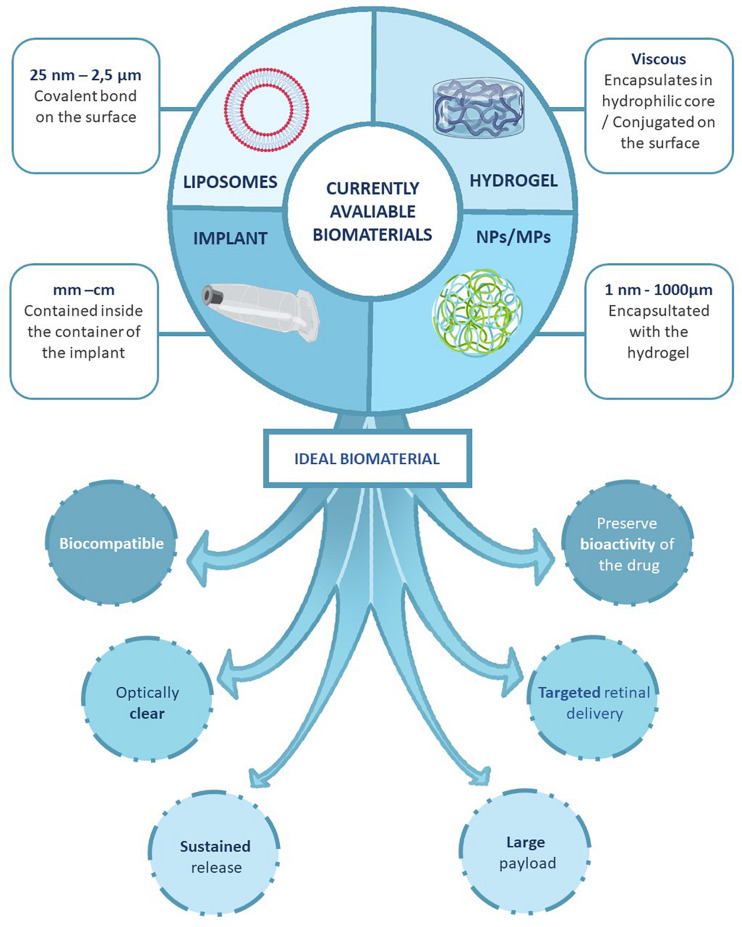
Developing strategies for the sustained drug delivery to the retina. We describe the type of systems that can be used to encapsulate the different molecules can be classified based on the size. The nano-formulations allows the encapsulation of the drugs directly to a molecule that favors the administration and the survival of the substance. The bulk systems protect the drug and allows a progressive administration of the substance during longer periods of time. And, we also represent the ideal characteristics of a sustained drug delivery platform for AMD.

**FIGURE 4:**
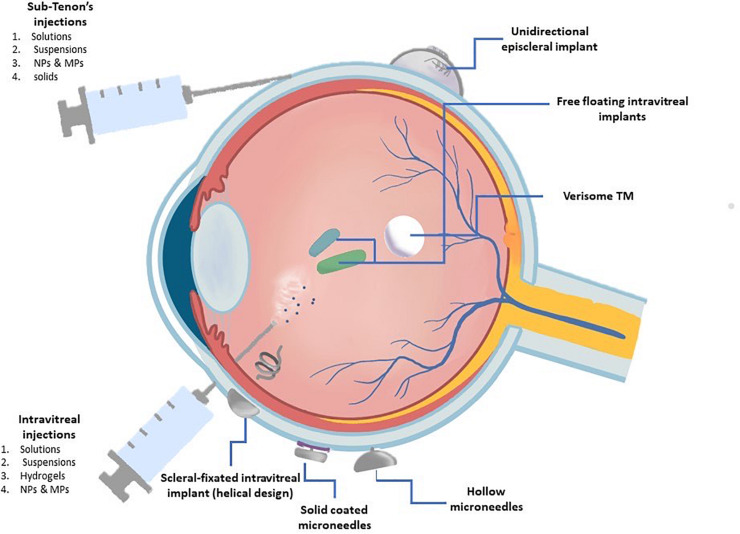
Representation of the ocular drug delivery methods discussed on this review. The treatment of retinal diseases is challenging due to the anatomic barriers and physiological clearance mechanism of the eye. This figure represents the methods currently used in the clinical setting to treat posterior segments diseases classified by their administration route.

Advances in biomaterial engineering and nanotechnology have fueled a growth of the research in prolonged DDD, made by biodegradable microparticles and nanoparticles, hydrogels or eye implanted devices, making them an attractive alternative to the frequent IVT injections (Kang-Mieler et al., 2020). Optimal biomaterials for sustained retinal drugs supply must meet the following properties [Figures 3, 4 (Seah et al., 2020)]:

• Be able to preserve the bioactivity of the molecule, protecting its tertiary and quaternary structure from denaturation, changes in pH or enzymatic degradation.
• Being able to deliver the molecules to the retina.
• Avoid raising intraocular pressure during administration. The biomaterial should be able to encapsulate a large amount of the drug in a minimal volume; in the case of an anti-VEGF, that means 0.5–2.0 mg of drug within a maximum volume of 0.05–0.1 ml.
• After inoculation, it should be able to maintain molecules’ release for >1 month, thus avoiding frequent administrations.
• Be biocompatible and remain optically transparent within the vitreous humor to avoid any interference with vision.

### Drug Delivery Devices (DDD)

Ganciclovir, for retinitis treatment by cytomegalovirus, was the first FDA-approved implantable DDD. With this implant, controlled release rates were obtained, which maintained active drug concentrations below toxic levels, while achieving high drug concentrations with limited systemic side effects (Yasukawa et al., 2004; Ebrahim et al., 2005). With the success of this implant, numerous biodegradable and non-biodegradable devices have been developed and many of them are in clinical use (Kang-Mieler et al., 2020). Implantable devices aim at providing structural/mechanical support for the localization of the drugs in the eyeball and to function as vehicles for controlled releases. Such DDD potentially locate therapeutic agents in the vitreous, lowering systemic exposure and, thus, reducing possible toxicity and/or side effects (Lim et al., 1999; Bourges et al., 2006; Kang-Mieler et al., 2020). They have many advantages over the more traditional administration methods since they improve the convenience, safety and efficacy of pharmacological therapies, bypass BRB and allow the sustained release of the drug directly at the target site, while decreasing the risk of infection or retinal detachment.

Drug delivery devices can be either biodegradable [e.g., PCL, PLA, PGA, PLGA, among others; or non-biodegradable (e.g., silicone), the latter needing to be surgically removed post use]. Ordinarily, implants are placed in the vitreous cavity by surgical intervention, although newer models are placed periocularly (e.g., transsclerally or suprachoroidally) or by IVT injection (del Amo and Urtti, 2008; Gaudana et al., 2009; Patel et al., 2013; Journal et al., 2018). Table 4 show currently available devices, the majority of them still in trial stages.

**TABLE 4:** Anti-VEGF intravitreally injectable and surgically implantable devices. **TABLE 4.1 |** Anti-VEGF intravitreally injectable devices (AVEGF-IID).

Product	Active principle	Type	Description	Stage/TRL	Advantages	Disadvantages	Duration
Iluvien (Alimera Science) (Jaffe et al., 2000; Kane et al., 2008; Schmit-Eilenberger, 2015)	Fluocinolone acetonide	Silicone and PVA	Silicone and PVA membrane polyimide tube, encapsulating 0.19 mg fluocinolone acetonide	FDA approved (2014, for DME)	Long lasting drug delivery, drastically decreases injections frequency	Non-biodegradable, needs surgical removal, provokes high IOP and cataract, contraindicated in glaucoma	36 months
Ozurdex (Allergan) (Haller et al., 2010; Blumenkranz et al., 2011; Boyer et al., 2011; Bezatis et al., 2013; Querques et al., 2013; Whitcup and Robinson, 2015)	Dexamethasone	PLGA	PLGA drug delivery device containing micronized 0.7 mg dexamethasone	FDA approved (2009). EMA approved (2010)	Biodegradable	Contraindicated in presence of glaucoma, risk of implant migration to the anterior chamber	6 months
Brimonidine implant (Allergan) (Burke and Schwartz, 1996; Gao et al., 2002)	Brimonidine tartrate	Polymer matrix	Polymer matrix containing Brimonidine tartrate (alpha-2- selective adrenergic receptor agonist)	Phase II	Biodegradable	Limited amount of drug	6 months
ENV705 (Envisia Therapeutics) (Benjamin et al., 2014; Navratil et al., 2014)	Bevacizumab	PLGA	PLGA-made implant containing bevacizumab/trehalose; fabricated with PRINT technology	Pre-clinical rabbit	Biodegradable	Release kinetics often not ideal, risk of migration due to lack of fixation	6 months
Nano-porous film device (Desai and Bernards, 2014; Lance et al., 2015)	Ranibizumab	PCL nanopori	PDMS-made PCL/nanoporous film	Pre-clinical rabbit	Biodegradable	No serious side effects	4 months

*Biomaterials-based solid implantable degradable devices. Principal characteristics, advantages and disadvantages. PVA, poly vinyl alcohol; IOP, increased intraocular pressure; PLGA, polylactic-co-glycolic acid; FDA, US Food and Drug Administration Agency; DDD, drug delivery device; DME, diabetic macular edema; PCL, polycaprolactone; PDS, port delivery system; PDMS, polydimethylsiloxane.*							

**TABLE 4.2 |** Surgically implantable devices.							

**Product**	**Active principle**	**Type**	**Description**	**TRL**	**Advantages**	**Disadvantages**	**Duration**

PDS (Genetech/For Sight Vision 4) (Campochiaro et al., 2019)	Ranibizumab	PDS	*Trans*-scleral implantable device	Phase II	Long-lasting delivery, minimal to no burst or lag time, constant zero-order release, release controlled by diffusion	Non-biodegradable, surgically implanted and removed	Refillable
Microelectro-mechanical system (MEMS-based devices) (Lo et al., 2009; Humayun et al., 2014)	Any drug	PDMS	Flexible cannula forming a refillable reservoir, implantable sub-conjunctival	Humans	Variable delivery rates, reduces complications with repeated intraocular injections	Non-biodegradable, surgically implanted and removed	Refillable 3 m study

*Biomaterials-based solid implantable refillable devices. Principal characteristics, advantages and disadvantages. PDS, port delivery system; PDMS, polydimethylsiloxane.*

#### Intravitreally Injectable Devices

Durasert^TM^, an EyePoint Pharmaceuticals (Watertown, MA, United States) is a 3.5 mm-long and Ø 0.37 mm solid polymer implant, IVT injectable with a small gauge needle and capable of up-to 3 years release of small molecules. It allows customization of release duration, linear release kinetics, and high drug loading (Callanan et al., 2008; Haghjou et al., 2011; Kang-Mieler et al., 2020). Durasert^TM^ gave origin to two different versions for the administration of Fluocinolone acetonide (Iluvien^®^) and Dexamethasone (Ozurdex^®^), as a plausible treatment for macular pathologies. Iluvien (Alimera Sciences, 2019) is a small non-biodegradable silicone implant to diffuse a low dose of fluocinolone acetonide (0.23–0.45 μg/day) for 18–36 months through a PVA capsule. The implant has to be removed surgically, it increases intraocular pressure, provokes cataract and is contraindicated for glaucoma patients (Jaffe et al., 2000; Kane et al., 2008; Ghasemi Falavarjani, 2009; Kuno and Fujii, 2010; Schmit-Eilenberger, 2015; Mandal et al., 2017; Alimera Sciences, 2019; ClinicalTrials.gov, 2020d, f). Iluvien was approved by the FDA in 2014 for the treatment of diabetic macular edema and by the United Kingdom Agency in 2012 for chronic diabetic macular edema-associated visual impairments (ILUVIEN)^1^. Ozurdex (Allergan) is a biodegradable poly lactic-co-glycolic acid (PLGA) solid implant which delivers dexamethasone. It permits long-lasting drug delivery, but it has serious collateral effects, like increased intraocular pressure, cataract formation and uveitis, due to the side effects of the corticosteroids and to the traction exerted to the vitreous humor. This system is contraindicated in patients with glaucoma. The device was approved by the FDA in 2009 and by the EMA in 2010 (Kuppermann et al., 2007; Haller et al., 2010; Kuno and Fujii, 2010; Blumenkranz et al., 2011; Boyer et al., 2011; Bezatis et al., 2013; Querques et al., 2013; Whitcup and Robinson, 2015; Mandal et al., 2017).

Brimonidine (Allergan) is a biodegradable polymer matrix containing Brimonidine tartrate (alpha-2-selective adrenergic receptor agonist). This molecule is normally used in glaucoma but recent studies suggest it also provides protection of retinal cells from degeneration in AMD geographic atrophy (Burke and Schwartz, 1996; Gao et al., 2002; Ghasemi Falavarjani, 2009; Campochiaro et al., 2012; Lai, 2013). Brimonidine is efficient and lacks toxicity, however, the amount of drug that can be loaded is limited because of the size of the molecule. Additionally, the hydrophilic nature of the molecule limits the sustained-release possibilities. The drug has achieved Phase II clinical trials (ClinicalTrials.gov, 2020c).

ENV705 (Envisia Therapeutics) is an IVT injectable biodegradable implant made with PGA and fabricated with PRINT technology, which provides a sustained release of Bevacizumab for up to 6 months. This implant is capable of releasing effective therapeutic drug concentration, but the release kinetics are often not ideal. The drug is currently in preclinical trials (Benjamin et al., 2014; Navratil et al., 2014).

Zordera is a Ø 10 mm biodegradable nano-porous cylindric device for long-lasting sustained release of Ranibizumab. Made by sandwiched PCL films with millions of porous of the molecule size of the drugs (Bernards et al., 2012; Schlesinger et al., 2015). The device is IVT implanted by a syringe (Desai and Bernards, 2014; Lance et al., 2016). The device is still in preclinical phase (Radhakrishnan et al., 2017; Mandal et al., 2018).

#### Surgically Implantable Devices

Subconjunctivally implantable pumps are versatile and affordable alternatives, permitting long-lasting, sustained and well controlled drug releases that avoid the need for repeated surgical interventions. Pumps consist of a small reservoir connected to a cannula which is inserted through the sclera into the eye, and they are empowered with a valve to control both, number of the doses and doses volume, thus optimizing and personalizing drug release profiles. They are refillable through a thin needle.

Port Delivery Systems, PDS (Genentech/For sight Vision4, Inc.) is a semipermeable, non-biodegradable refillable transscleral port delivery system, surgically implanted through a 2–3 mm incision and fixed to the sclera to avoid migration. The device has been used for long-lasting sustained release of Ranibizumab. Because of the sclera surface this device permits easy and low invasive transscleral drug delivery with increased absorption rate. However, serious adverse effects were reported, including endophthalmitis and persistent vitreous hemorrhage (Mainardes et al., 2005; Shah et al., 2010; Kim et al., 2017; Mandal et al., 2017; Campochiaro et al., 2019). Phase II clinical trials showed that 15 months recharge frequency with drug concentration of 100 mg/ml have equivalent visual and anatomical effects to patients treated with monthly IVT ranibizumab injections (Campochiaro et al., 2019). Also, implant insertion and reloading procedures were well tolerated by patients. The system is now under Phase III trials, expecting to reach 3 years implants with recharges every 6 months: PORTAL (NCT03683251) and ARCHWAY (NCT03677934) trials, expected to end in January 2022 and April 2022, respectively (ClinicalTrials.gov, 2020b, e).

Replenish^®^ is a sub-conjunctival refillable, non-biodegradable MEMS for long-lasting sustained drug release. It is a sub-conjunctively placed PDMS 0.6 ml refillable reservoir with a flexible cannula and a valve. The cannula is inserted through the sclera and fixed with sutures (Lo et al., 2009). It is refillable up to 10 times with a thin needle. Replenish, Inc., was planning to start Clinical trials for AMD treatment (Lo et al., 2009; Gutiérrez-Hernández et al., 2014; Humayun et al., 2014).

### Injectable Hydrogels

To overcome some of the limitations of implantable DDD and to avoid surgeries, injectable hydrogels have emerged as an alternative for IVT drug administration using different biomaterial formats.

A hydrogel is a three-dimensional network of hydrophilic polymers that can swell in water and retain a large amount of H_2_O molecules while maintaining its solid state (Li and Mooney, 2016). Despite the high solubility of hydrophilic polymers (in water), hydrogels resist dissolution and even interact favorably with H_2_O molecules, because their hydrophilic polymer chains form a strongly crosslinked network (Li and Mooney, 2016). Their high H_2_O percentage (>10% of the total weight) make them easy-to-manipulate, flexible materials. Furthermore, their porous structure makes them suitable for encapsulation of drugs, biomolecules, or even stem cells. Hydrogels are highly biocompatible and can undergo a volume phase, or gel-sol phase transition, either spontaneously after injection (*in situ*-forming hydrogels) or triggered by specific external stimuli (changes of pH, temperature, pressure, light intensity, etc.) (Bahram et al., 2016; Chang et al., 2019). Injected into the eye, hydrogels provide both, structural support and long-term sustained release of the incorporated drugs, molecules or stem cells’ secretome (Alexander et al., 2014; Kirchhof et al., 2015a; Su et al., 2015; Yu et al., 2015; Seah et al., 2020).

The employed polymers can be natural, synthetic or hybrid. Natural polymers (cellulose, chitosan, alginate, hyaluronic acid, silk fibroin, etc.) are degraded by the enzymes of the body, so their biodegradation rate cannot be adjusted (Lovett et al., 2015; Yu et al., 2015). Synthetic polymers (PCL, PEG, PLGA, PLLA, NIPAAm, etc.) properties such as porosity, swelling capacity, stability, mechanical resistance and biocompatibility can be adjustable by varying their chemical composition and preparation methods. Within this group, we find both biodegradable polymers such as PEG (Anwary et al., 2018), PLGA (Arranz-Romera et al., 2019), etc. which can be degraded by the action of biological factors such as changes in pH, increase in temperature, enzymatic activity or by the immune system itself; and non-biodegradable polymers such as PDMS (Pirmoradi et al., 2011; Humayun et al., 2014). Hybrid polymers display the characteristics of their individual components (Yu et al., 2015, 2019; Osswald and Kang-Mieler, 2016; Awwad et al., 2019). Incorporation of therapeutic agents (e.g., anti-VEGF drugs) to a hydrogel strongly depends on the crosslinking of gel’s polymers. Molecules incorporation to a covalently crosslinked gel requires chemical actions, which often result in modification and/or inactivation of the bioactive molecule as well as in increased toxicity (Kang Derwent and Mieler, 2008; Matanović et al., 2014; Awwad et al., 2018, 2019; Seah et al., 2020). On the other side, molecules incorporation to a non-covalently crosslinked gel needs an *ad hoc* tuning of the manufacturing parameters, so important characteristics of the gel (e.g., gelation time and diffusion rate) become difficult to predict (Seah et al., 2020).

Injectable hydrogels allow reducing drug administration frequency and, even more important, avoid eye procedures-associated complications, including blurred vision or irritation However, hydrogels require IVT administration, show poor penetrability and specificity, and present difficulties for correct sterilization (Alexander et al., 2014; Kirchhof et al., 2015a; Su et al., 2015; Yu et al., 2015; Galante et al., 2018; Seah et al., 2020).

Several hydrogels have been used for drug delivery in different non-ocular pathologies, with favorable results (Sharpe et al., 2014). Despite their theoretical advantages, hydrogels for the ocular route face serious difficulties to meet safety, tolerability, manufacturability, degradability, and facility of administration requirements, while maintaining the bioactivity and clinical effectiveness of the desired drug (Chang et al., 2019). To our knowledge, no hydrogel has been approved by FDA or EMA and only one of them has reached clinical trials stage (Ocular Therapeutix), all other being still in a preclinical stage, Table 5).

**TABLE 5:** Injectable hydrogels.

Product	Active principle	Description	Model	Gelation time	Advantages	Disadvantages	Release
Hyaluronic acid/Dextran (Yu et al., 2019)	Bevacizumab	VS-HA chemically crosslinked to a Dex-SH *in situ*-forming hydrogel	Monkey	30 s	Non-toxic, transparent, biocompatible, good mechanical strength, easy functionalization	Initial burst release	5–6 months
Silk Fibroin (Lovett et al., 2015)	Bevacizumab/Ranibizumab	Crosslinked silkworm silk fibroin	Rabbit	Modifiable	Non-toxic, biocompatible, good mechanical strength, controllable degradation, sustained drug release rate	Initial burst release	∼90 days
ESHU (Rauck et al., 2013)	Bevacizumab	PU coupled with PEG	Rabbit	3 m at 37°C	Thermo-responsive polymer, no initial burst release, no significant inflammation	–	9 weeks
OTX-TKI (Jarrett et al., 2017)	Tyrosine kinase inhibitor	Micronized TKI particles in hydrogel	Rabbit	–	Bioabsorbable, safe, sustained drug release and tolerability	–	24 weeks
(PEOzPCL-PEOz) copolymer (Wang et al., 2012)	Bevacizumab	(PEOzPCL-PEOz) copolymer thermosensitive biodegradable hydrogels	Rabbit	60 s	Reversible sol-gel transition, intraocular biocompatibility, extended drug release rate	Low initial drug concentration (loading dose)	8 weeks
mPEG-PLGA-BOX (Hu et al., 2019)	Bevacizumab	Thermosensitive polymer mPEG-PLGA with BOX as linker	Rabbit		No initial burst release. Biocompatibility and reduction of angiogenesis.	–	5 weeks
EPC (Xue et al., 2019)	Bevacizumab/Aflibercept	Polymer synthetized from PEG, PPG and PCL diol using HMDI as coupling agent	Rabbit	–	Thermo-responsive polymer.	Initial burst release	4 weeks
PNIPAAm/PEG-DA (Alexander et al., 2014; Drapala et al., 2014)	Bevacizumab/Ranibizumab	Cross-linking PNIPAAm with PEG-DA thermosensitive hydrogel.	Rat	Once injected	Synthetic, biocompatible, thermo-responsive polymer, good mechanical strength	Initial burst release. No improvement	<4 weeks
(PEOzPCL-PEOz) copolymer (Wang et al., 2012)	Bevacizumab	(PEOzPCL-PEOz) copolymer thermosensitive biodegradable hydrogels	*In vitro*	60 s	Thermosensitive reversible sol-gel transition. 90% viability of cultured cells	Low initial drug concentration (loading dose)	8 weeks
NIPAAm/Ac-HA (Awwad et al., 2019)	Bevacizumab	Cross-linking NIPAAm gel with Ac-HA	*In vitro*	–	Thermo-responsive polymer, sustained drug release	Initial burst release	7 weeks
EPC (Xue et al., 2019)	Bevacizumab/Aflibercept	Polymer synthetized from PEG, PPG and PCL diol using HMDI as coupling agent	*In vitro*	–	Thermo-responsive polymer. Pharmaco-hydrogel inhibits the growth of angiogenic cells	Initial burst release	6 weeks
Diels-Alder hydrogel (Kirchhof et al., 2015b)	Bevacizumab	PEG macro-monomer chemically crosslinked by Diels-Alder reaction	*In vitro*	14–171 m	Good mechanical strength, controllable degradation, sustained drug release rate	Initial burst release	<6 weeks
PEG thiol/maleimide (Yu et al., 2014)	Bevacizumab	*In situ*-gelling hydrogel chemically cross-linked by thiol-maleimide reaction	*In vitro*	95–210 s	Predictable degradation, modifiable release rate by crosslinking density	Initial burst release	2 weeks
PEG thiol/maleimide (Santhanam et al., 2016)	Bevacizumab	*In situ*-gelling hydrogel chemically cross-linked by thiol-maleimide reaction	*In vitro*	90–120 s	Predictable degradation	Initial burst release	2 weeks
mPEG-PLGA-BOX (Hu et al., 2019)	Bevacizumab	mPEG-PLGA with BOX as linker	*In vitro*	–	No initial burst release, thermo-responsive polymer. Non-toxic		<2 weeks
Alginate-Chitosan (Xu et al., 2013)	Bevacizumab	Polysaccharides cross-linked hydrogel	*In vitro*	2 m at 37°C	Transparent, tailored degradation rate, modifiable release rate	Initial burst release	3 days
Hyaluronic acid/Dextran (Yu et al., 2015)	Bevacizumab	VS-HA chemically crosslinked to a Dex-SH *in situ*-forming hydrogel	*In vitro*	30 s	Non-toxic, transparent, and cytocompatibility. 99%Cytocompatibility of hydrogel	Initial burst release	1 day

*In vitro and in vivo trials of hydrogels-integrated injectable drugs. VS-HA, vinylsulfone functionalized glycosaminoglycan hyaluronic acid; Dex-SH, thiol-functionalized polysaccharide, dextran; PNIPAAm, Poly N-isopropylacrylamide; PEG-DA, poly ethylene glycol diacrylate; Ac-HA, acrylated hyaluronic acid; PEG, poly(ethylene glycol); mPEG-PLGA-BOX, methoxy-poly (ethylene glycol)-block-poly (lactic-co-glycolic acid); ESHU, poly(ethylene glycol)-poly-(serinol hexamethylene urethane); PU, polyurethane; EPC, poly(ether ester urethane); PPG, poly(propylene glycol); PCL, poly(ε-caprolactone); PEOzPCL-PEOz, poly (2-ethyl-2-oxazoline)-b poly (ε-caprolactone)-b-poly (2-ethyl-2-oxazoline). APRPG, liposomes modified with Ala-Pro-Arg-Pro-Gly. SU5416, 3-([2,4-dimetilpirrhol-5-il] metilidenil)-indolin-2-ona. SFN, silk fibroin nanoparticles. Bev-MVL, Bevacizumab-loaded multivesicular liposomes. OCCNP, oligochitosan coated cerium oxide nanoparticles.*

Using a bioabsorbable hydrogel (OTX-TKI), Ocular Therapeutix (Bedford, MA, United States) developed a TKI implant, capable to supply TKI for up to 12 months (Jarrett et al., 2017). OTX-TKI is under Phase I clinical study in Australia, to test its safety, durability and tolerability (Eyewire News, 2020). The same company in collaboration with Regeneron (Tarrytown, NY, United States), is developing an injectable version of OTX-IVT to provide sustained aflibercept release for 4–6 months (OTX-IVT, 2020).

Poly (2-ethyl-2-oxazoline)-b poly (ε-caprolactone)-b-poly (2-ethyl-2-oxazoline) (PEOzPCL-PEOz) copolymer-based thermosensitive biodegradable hydrogels for prolonged releases of bevacizumab were shown to be biocompatible *in vitro* and *in vivo* with a human retinal pigment cell line and in a lagomorph model, respectively, for 2 months (Wang et al., 2012).

Hydrogels of PEG synthesized via thiol-maleimide reaction from PEG-Mal and PEG-SH, loaded with bevacizumab did not generate cytotoxicity after 7 days of incubation and maintained a sustained drug release for 14 days *in vitro*, reaching 70% release in this time interval (Yu et al., 2014). Maintenance of drug bioactivity was not tested.

Long-term bevacizumab release from a ESHU hydrogel *in vivo* was well tolerated, without inflammation and without intraocular pressure alterations in rabbit retinas for 9 weeks. ESHU maintained continuous release with no initial burst and drug concentrations 4.7 times higher than free bevacizumab injections (Rauck et al., 2013).

A thermosensitive poly (*N*-isopropylacrylamide) hydrogel (PNIPAAm) made by crosslinking PNIPAAm with poly (ethylene glycol) diacrylate (PEG-DA) or poly (ethylene glycol diacrylate)-co-(L-lactic acid) (PEG-PLLA-DA) was capable of locally releasing bevacizumab or ranibizumab for 1 month, without inducing long-term effects on retinal function (Turturro et al., 2011; Drapala et al., 2014).

Silk fibroin hydrogels were studied for bevacizumab sustained delivery in a rabbit model for 3 months showing higher concentrations in day 90 than those in day 30 after direct injections and the gels started to biodegrade after 3 months (Lovett et al., 2015).

Yu et al., 2015 assessed the biocompatibility and release of bevacizumab of an *in situ* gelling hydrogel made by thiolated functionalized hyaluronic acid and thiolated dextran (HA-VS/Dex-SH) maintained relevant therapeutic concentrations, over 100 times higher than the freely injected drug, 6 months after injection and without inducing pathologies, in a lagomorph model (Yu et al., 2015). In primates, no cytotoxic effects were observed 21 weeks post-injection (Yu et al., 2019).

A thermosensitive hydrogel of methoxy-poly (ethylene glycol)-block-poly (lactic-co-glycolic acid) (mPEG-PLGA-BOX) released bevacizumab, with no burst release nor cytotoxicity and maintaining drug activity during 35 days in a rabbit model (Hu et al., 2019).

NIPAAM and Ac-HA hydrogel combined with bevacizumab was performed in the PK-Eye^TM^ model, an *in vitro* ocular flow model developed by Awwad et al. (2015) that simulates the times protein clearance at the posterior chamber of the human eye. This hydrogel formulation bevacizumab release for at least 50 days, maintaining a therapeutic dose in the posterior cavity of the eye model and with zero order kinetics post 5 days (Awwad et al., 2019).

Bevacizumab and aflibercept have also been encapsulated in a multiblock EPC thermoresponsive hydrogel consisting of PEG, poly (propylene glycol) (PPG) and poly(ε-caprolactone) (PCL) (Xue et al., 2019). Both anti-VEGF were released similarly and almost linearly for up to 40 days, remaining bioactive in a HUVECs cellular line model *in vitro*, as well as inhibiting vessel outgrowth in rat *ex vivo* choroidal explants. In an *in vivo* retinal neovascularization rabbit model, aflibercept anti-angiogenic bioactivity was maintained for 28 days and animals showed a reduction of vascular leakage.

An injectable polysaccharide cross-linked hydrogel (Alginate-Chitosan) was developed for Bevacizumab delivery and tested *in vitro*. It achieved a 3-days sustained with 4-h initial bursting (Xu et al., 2013).

Diels-Alder hydrogels synthesized and functionalized with [furyl (-Fur) and maleimide groups (-Mal)] for bevacizumab delivery showed >6 weeks *in vitro* release (Kirchhof et al., 2015b).

### Colloidal Particles

Colloidal systems (liquid suspensions with nanometric carriers) including nanosuspensions, micro/nanoparticles, liposomes, dendrimers and hydrogels have been postulated as promising alternatives for AMD therapy. They are micro/nanosized particles, based on either natural or synthetic materials and composed of highly stable lipophilic, hydrophilic or amphiphilic molecules, loaded with drug molecules (Kandatsu et al., 2005). These particles improve drugs solubility and act as reservoirs for long-term sustained release or for enhanced transport of proteins through the eye barriers (Hora et al., 1990; Herrero-Vanrell and Refojo, 2001; Medina et al., 2009; Li, 2012; Varshochian et al., 2013, 2015; Yandrapu et al., 2013; Ye et al., 2015; Imperiale et al., 2018). Such particles are usually composed of PLA, PLLA, PLGA, PEG, PEG-DA, alginate-chitosan and PLV and are injected IVT; being completely degradable, they do not require subsequent extraction surgery (Mi et al., 2002; Kang-Mieler et al., 2020).

Micro/nanoparticles capacity for tissue penetration, protection capabilities against proteolysis and prolonged drug-release periods depend on the characteristics of their external layer, which in turn depend on the polymer these micro/nanoparticles are made of Sakurai et al. (2001), Sahoo et al. (2008), Honda et al. (2013) and Seah et al. (2020).

Fluorescein-labeled polystyrene micro/nanoparticles studies in a lagomorph model showed that IVT microparticles are transported to the vitreous cavity and to the trabecular meshwork, while nanoparticles are transported to the retinal tissue (Sakurai et al., 2001).

Micro/nanoparticles provide several benefits as drug carriers for posterior ocular drug delivery, but the main one is that they are capable of increasing drug penetration across the conjunctival and corneal epithelia by temporarily altering the tight junctions when administered topically (Hans and Lowman, 2002). In addition to their ability to increase *trans*-corneal penetration, the ability to improve solubility of lipophilic drugs may play an important role in increasing IVT half-life and thus bioavailability of lipophilic drugs. This approach also provides sustained release of the encapsulated molecules. In cases with low encapsulation efficiency where it is difficult to establish or control the conditions of drug release, the systems can be modified for tissue-specific uptake and to protect the therapeutic molecules from degradation. Despite its advantages, there are limitations associated with formulation stability, high initial burst release, protein denaturation, clearance by the immune system, control of particle size, control of drug release rate and large-scale manufacturing of sterile preparations (Sakurai et al., 2001; Hans and Lowman, 2002; Kang-Mieler et al., 2020; Seah et al., 2020). Still, many of these particles are being evaluated preclinically for the administration of drugs into the posterior segment of the eye as carriers of anti-VEGF factors for AMD.

Intravitreal injections of PLGA microspheres-encapsulated bevacizumab in a lagomorph model showed higher concentrations in the vitreous and aqueous humor than of freely-injected drug, and a maintenance of their pharmacological activity for over 42 days (Ye et al., 2015). However, authors did not determine drug’s ability to reach the target tissue, neither its long-lasting effects.

PLGA-albumin nanoparticles for sustained supply of bevacizumab, made from a double water-in-oil-in-water emulsion showed bioactive IVT concentrations (>500 ng/ml) for 8 weeks after IVT injection in a rabbit model (Varshochian et al., 2015). More recently, PLGA nanospheres-encapsulated fenofibrate showed full biocompatibility and attenuation of both, VEGF expression and vascular tissue disruption for up to 60 weeks after IVT injection in a rat W-AMD model, a diabetic retinopathy rat model and a very low-density lipoprotein receptor knockout (Vldlr–/–) mouse model (Qiu et al., 2019). Despite PLGA’s good biodegradability and biocompatibility, one of the main problems is that PLGA degeneration causes an accumulation of lactic and glycolic acid in the sphere, which provokes a decrease of the pH and a consequent denaturation of the drug (Liu et al., 2019a, b; Qiu et al., 2019). A possible solution to this problem is the manufacture of monomer conjugated PLGA microspheres which prevent rapid degradation of the biomaterial and preserve the biocompatibility of the drug for a longer time.

Silk fibroin abilities as nanocarrier for anti-VEGF particles has been tested by encapsulating bovine serum albumin (BSA, macromolecular model with similar weight to the antibodies that are usually used in AMD) showing a prolonged retention compared to free BSA solution and better distribution in *in vitro* studies with ARPE-19 and *in vivo* studies in a rabbit model (Yang et al., 2019).

The biggest limitation of the use of micro/nanoparticles is the small amount of drug that they are able to encapsulate inside, therefore, prolonged treatments require frequent IVT injections that can cause discomfort to the patient and even eye damage during the procedure (Seah et al., 2020).

Another colloidal system used today are liposomes (Abrishami et al., 2009; Shah et al., 2010; Patel et al., 2013; Davis et al., 2014; Ameeduzzafar et al., 2016), vesicles consisting of a phospholipid bilayer membrane incorporating water-soluble and lipid-soluble drugs in aqueous and lipid phases, respectively. Compared to nanoparticles, liposomes usually have low immunological reactivity or toxicity since phospholipids are easily metabolized once the liposome is degraded (Seah et al., 2020).

These vesicles provide sustained release of drugs with decreased frequency of dosing. The systems also allow for modifications of the rate of drug release and the ability to provide stimulus-sensitive drug release. However, these vesicles present some limitations including blurred vision after injection of the liposomal suspension into the vitreous body, low reproducibility, instability of the macromolecules during production, variable size distribution and storage conditions specific to the composition of the drug and liposome.

Numerous animal studies have shown that IVT injected liposomal drugs release their aqueous contents slowly, protecting the intercalated substances from degradation and clearance. This process avoids the toxicity from the high peak concentrations normally seen after injection of free drug. Therefore, the liposomal drug release formulations may lead to greater clinical efficacy by maintaining therapeutic concentrations for longer time intervals. Liposomal applications in the posterior segment provide longer clearance times, less toxicity, and specific delivery.

Furthermore, the surface of the liposomes can be functionalized to improve penetration through the different tissues and to reach the target tissue. In a study by Davis et al. (2014), the liposome surface was modified using annexin-A5 with the aim of improving the administration of Bevacizumab through the epithelial barriers of the cornea (Davis et al., 2014). This study verified that annexin functionalization allowed liposomes to reach the retina more easily (however, the results still require improvement since only 1% of the dose reached the target).

In other studies, such as Honda et al. (2013) in which the effects of Semaxanib-SU5416 (VEGF receptor proteinTKI, an angiogenesis inhibitor) were evaluated, it was demonstrated that the use of liposomes allowed to decrease choroidal neovascularization (CNV) activity in animal model compared to the control group. That is, liposomes allowed the drug to reach the target tissue more easily than the drug alone (Honda et al., 2013).

Studies have shown that the release of anti-VEGF drugs in liposomes can be maintained for more than a month without causing cytotoxicity (Mu et al., 2018). In the latter study, they demonstrated that changing the aqueous/lipid ratio improved the encapsulation efficiency of the drug (Bevacizumab) in Brown-Norway rats with laser-induced choroidal neovascularization. IVT liposome-treated eyes with bevacizumab in rabbits maintained high drug levels for >56 days.

The limitations that exist in the use of liposomes as a drug transport system must be taken into account. One of the main limitations of this colloidal system is the lack of studies that exist today. Drug release from liposomes to target tissue is highly dependent on environmental conditions such as pH or enzyme activity (Villegas et al., 2017). Because there are so many factors that can affect liposome stability, it is difficult to predict drug release. Therefore, although liposomes have structural characteristics that allow effective administration of the drug and degradation of the transport system that does not cause toxicity, a more exhaustive study of the environmental factors that cause the degradation of liposomes is necessary.

A slightly different device is Verisome IBI-20089 (Icon bioscience), actually in Phase I/II clinical trials (ClinicalTrials.gov, 2020a), consisting of a biodegradable non-polymer injectable liquid (carbonates, tocopherols, and citrate ester) that forms small spherules once injected into the vitreous. It has been formulated to deliver intravitreal injections of triamcinolone acetonide (TA) in combination with ranibizumab in W-AMD for >1 year, showing good safety and efficacy (Lim et al., 2015).

Table 6 summarizes some of the colloidal systems (micro/nanoparticles and liposomes) for AMD therapies.

**TABLE 6:** Injectable colloidal particles.

Product	Active principle	Format	Description	Model	Advantages	Disadvantages	Release
Verisome IBI-20089 (Icon Bioscience) (Lim et al., 2015)	Triamcinolone acetonide + Ranibizumab	Small spherules	DDD that forms small spherules once injected into the vitreous	Phase I/II	Biodegradable, decreases injection frequency	Damages the choroid and destroys retinal layers (at high densities)	<12 months
PLGA-Albumin NPs (Varshochian et al., 2015)	Bevacizumab	NP	Fabricated by w/o/w double emulsion in presence of albumin as a stabilizer. Particle size, 197 nm	Rabbit	Stable, sterile	Initial burst release	16 weeks
PLV (Abrishami et al., 2009)	Bevacizumab	Liposome	EPC-Chol and DPC-Chol liposomes formed by dehydration and rehydration followed by freeze drying	Rabbit	Prolonged drug residency	No demonstrated efficiency	6 weeks
Anexine 5 on PLV (Davis et al., 2014)	Bevacizumab	Peptide-conjugated liposome	PC-PS-Chol-Toc liposomes made by dehydration and rehydration, coated with annexin. Particle size 100 nm	Rabbit	Enhanced bioavailability. Transscleral delivery	No demonstrated efficiency	1 week
PLGA MPs (Ye et al., 2015)	Bevacizumab	MP	Fabricated by solid-in-oil-in-hydrophilic oil. Particle size, 2–7 μm	Rabbit	–	Initial burst release	>2 weeks
SFN (Yang et al., 2019)	BSA	NP	Regenerated silk fibroin	Rabbit	Accumulated distribution and extended retention	Loading capacity might be limited by its physico-chemical properties	1–2 weeks
Bev-MVL (Mu et al., 2018)	Bevacizumab	Liposome (multivesicular)	Bev-MVLs with high encapsulation efficiency. Prepared by double emulsification technique	Rabbit/rat	Sustained release. High retention time in the vitreous	–	2 months
PLGA-NPs (Qiu et al., 2019)	Fenofibrato	NP	NP are prepared using an emulsification method with PLGA and fenobibrato fenofibrate. Particle Size, 250 nm	Mouse/rat	Sustained therapeutic effects. Prolonged release. Potentially reduced injection frequency	Need to optimize effective drug loading of Pheno-NP and release kinetics	32 months
APRPG (Honda et al., 2013)	SU5416	Liposome	Liposomes were prepared using the thin-film hydration method	Rat	Significant reduction of the CNV area	VEGF inhibition could affect normal and angiogenic vessels	1–2 weeks
Silica-based hydrogel (William et al., 2012)	Bevacizumab	NP	NP synthesized by electrochemical etching and oxidation of silicon wafer in hydrofluoric acid. Particle size, 100 nm	*In vitro*	Drug release by matrix erosion, can deliver any type of drug	Initial burst release, hydrogel formed in a syringe	∼12 months
PEG-PLLA-DA/NIPAAm (Liu et al., 2019b)	Ranibizumab	PLGA microspheres in NIPAAm crosslinked with PEG-PLLA-DA	Microparticles are synthesized and charged by double emulsion solvent evaporation technique	*In vitro*	Thermo-responsive polymer, modifiable release rate, sustained release	Initial burst release	6 months
Alginate-Chitosan/PLGA (Xu et al., 2013)	Bevacizumab/Ranibizumab	PLGA microspheres encapsulated into AC-H	Synthesis of different systems of hydrogel for drug release	*In vitro*	Biocompatible, controlled degradation rate, sustained drug release	Initial burst release	<6 months
PEG-PLA (Li, 2012)	Bevacizumab	MP	Fabricated by Double emulsion. Particle size, 2-10 um	*In vitro*	–	Initial burst release	3 months
mPEG-PLGA (Pan et al., 2011)	Bevacizumab	MP	mPEG-PLGA-made multiblock copolymer hydrogel by 2,2-bis 2-oxazoline. Particle size, 2–10 μm	*In vitro*	–	Initial burst release	1 month
PLGA (Pandit et al., 2017)	Bevacizumab	PLGA nanoparticles covered with chitosan/alginate	Bevacizumab loaded CS-coated PLGA NPs was prepared by double emulsion solvent evaporation method	*In vitro*	Stable and sustained drug release	–	3 days
OCCNP (Wang et al., 2018)	Cerium Oxide	NP/Alginate-hydrogel	Cerium oxide- oligo-chitosan NP/alginate-hydrogel that have antibacterial and anti-inflammatory functions	*In vitro*	Strong antioxidant, O2 radical absorption. Anti-inflammatory. VEGF inhibition	–	2 days

*In vitro and in vivo trials of colloidal particles-integrated injectable drugs. PLGA, polylactic-co-glycolic acid; AC-H, alginate-chitosan hydrogels; MP, microparticles; NP, nanoparticles; mPEG-PLGA: methoxy-poly (ethylene glycol)block-poly lactic-co-glycolic acid; EPC-Chol, egg phosphatidylcholine-cholesterol; DPC-chol, 1,2 dipalmitoyl-sn-glycero-3-phosphocholine; Ac-HA, acrylated hyaluronic acid; PEG, poly(ethylene glycol); PEG-PLLA-DA/NIPAAm, poly(ethylene glycol)-co-(L-lactic-acid) diacrylate/N-isopropylacrylamide; BSA, bovine serum albumin.*

### Compound Particles-Hydrogels Drug Delivery Technologies

Despite the already discussed advantages of injectable polymeric micro/nanoparticles, one of their main limitations is locating them at the injection site of the eye. A normal eye can eliminate microparticles in 50 days and vitrectomized ones in 14 days (Morirero et al., 1991). As a solution to limit the movement of these particles, the use of injectable particles-hydrogels has been proposed, to provide a prolonged and localized release of the drug after injection (Kang-Mieler et al., 2017). These compound systems offer advantages over each of the two platforms separately, by reducing the initial explosion and extending the release time (Osswald and Kang-Mieler, 2015, 2016).

A thermosensitive biodegradable hydrogel-microspheres fabricated by suspending, poly (lactic-co-glycolic acid) (PLGA) microspheres with ranibizumab within a poly (ethylene glycol)-co-(L-lactic-acid) diacrylate/*N*-isopropylacrylamide (PEG-PLLA-DA/NIPAAm) hydrogel, achieved a controlled release of ranibizumab and aflibercept for 6 months (Liu et al., 2019b, c), while efficacy was tested *in vivo* in a rodent choroidal neovascularization model (Osswald et al., 2017; Liu et al., 2019c).

The anti-angiogenic and anti-inflammatory properties of cerium oxide nanoparticles coated with oligo-chitosan in alginate hydrogel were studied in an *in vitro* model of human retinal pigment epithlium-19 (ARPE-19) and umbilical endothelial cell lines. Sustained nanoparticles release and controlled hydrogel degradation with strong antioxidant properties and reduction of apoptosis were observed for 2 months. Furthermore, in the same model, they suppress bacterial lipopolysaccharides-induced inflammatory responses, inhibit VEGF expression and reduced cell apoptosis (Wang et al., 2018).

### Externally Light-Triggered Drug Release Hydrogels or Nanoparticles

Extending the half-life of the therapeutic agent can reduce dosage, costs and injections frequency. Alternative strategies to increase the half-life of anti-angiogenic drugs after IVT injection without losing effectiveness include nanotechnology-based DDD, for example hydrogels, liposome, microsphere, micro/nanoparticles, etc. (Fuchs and Igney, 2017; Machinaga et al., 2018; Nayak and Misra, 2018). Nevertheless, none of them allows an external control of the timing of the release.

To overcome this drawback several photoactive biomaterials have been developed which release the drug after being exposed to an external ultraviolet (UV) or visible light stimulus. Light-responsive systems can be classified into three categories according to the light-triggering mechanism for drug release: (1) photochemical, where light breaks a covalent bond (2) photoisomerization, where light induces conformational changes, and (3) photothermal, where light produces heat after photoexcitation to selectively affect thermally modulated components of the system (Linsley and Wu, 2017). This is a well-known technology, mainly tested in cancer therapy, translated to posterior segment eye diseases due to the transparency of the cornea, which allow easy light stimulation of the back of the eye. Nevertheless, very few studies have been dedicated to AMD treatment (Table 7).

**TABLE 7:** *In vitro* and *in vivo* trials of light triggered biomaterials-integrated drugs.

Product	Active principle	Wavelength (nm)	Description	Model	Advantages	Disadvantages	Release
NP/UVSP (Huu et al., 2015)	Nintedanib	365	PLGA NP in light-degradable polymer DMN-based	Rat	Biocompatible NP and irradiation safe for the eye. Non-cytotoxic	Initial burst release	<30 weeks
NP-CPP (Wang et al., 2019)	Doxorubicin	400	PEG-PLA chains modified by CPP and binded to DEACM	Mouse	Biocompatible and irradiation safe for the eye. Non-cytotoxic	Initial burst release. >90% of the drug is released in 48h	1 week
Agarose-AuNPs (Basuki et al., 2017)	Bevacizumab	400–500	Polymer-functionalized gold nanoparticles in agarose hydrogel	*In vitro*	Modifiable release rates by changes in hydrogel composition	–	15 days (9 cycles)
NIPAM-AuNR (Jiang et al., 2019)	Doxorubicin and curcumin	808	AuNRs in polymerizated NIPAM with nanogels as crosslinker	*In vitro*	Lineal release of the drug in each irradiation cycle	–	70 months (10 cycles)

*NP, nanoparticles; UVSP, ultraviolet-sensitive polymer; PLGA, polylactic-co-glycolic acid; DMN, 4,5-Dimethoxy-2-nitrobenzyl; NP-CPP, nanoparticles modified with cell penetrating peptide; PEG-PLA, poly(ethylene oxide)-poly(D,L-lactic acid) block copolymer; DEACM, 7-(diethylamino)coumarin-4-yl]methyl carboxyl; AuNPs, polymer-functionalized gold nanoparticles; NIPAM, N-Isopropylacrylamide; AuNR, gold nanorod.*

We have identified two systems with photochemical and two with photothermal activation of drug release for AMD treatment.

A nanoparticle storage platform for on-demand drug delivery, based on a UV ultra-degradable polymer, which releases drug after a brief exposure to 365 nm UV light (Fomina et al., 2010; Huu et al., 2015). This polymer contains a fraction of *o*-nitrobenzyl in each monomer, responding to the absorption of UV by degrading into fragments and small molecules through quinone-methide rearrangements. The system releases drug for >30 weeks post-injection and authors reported light-induced release of Nintedanib, an angiogenesis inhibitor, as well as suppression of choroidal neovascularization in rats for >10 weeks (Huu et al., 2015).

A nanoparticles-based DDD which, injected intravenously, can release the drug into the eye by 400 nm light activation was developed and tested by Wang et al. (2019). Nanoparticles (NP-CPP) were composed of poly (ethylene oxide)-poly (D, L-lactic acid) (PEG-PLA) chains modified with Tat-C peptide (CPP), that allows their entry into cells. CPP is reversely binded by a covalent bond to a DEACM group that prevents entry into cells. After the exposure to light, DEACM releases CPP, which migrates from the center of the nanoparticle to the surface, activating it. Toxicity and biocompatibility of the system were tested both, *in vitro* and *in vivo*. Irradiation of the eyes after intravenous injection provoked NP-CPP accumulation in mouse neovascular lesions. Doxorubicin-loaded NP-CPP significantly reduced the size of the neovascular lesion, the majority of the drug being released 48 h post-irradiation.

Chromophores coupled with a thermally responsive drug-releasing materials gave origin to two different photothermal DDD (Basuki et al., 2017; Jiang et al., 2019).

The former is based on a photo-thermal interaction of polymer-coated AuNPs within an agarose hydrogel. Stimulation by 400–500 nm-light radiation increases local temperature which in turn causes a reversible softening of the hydrogel matrix and a consequent release of the drug. Release profile is adjusted by modifying AuNPs and agarose concentrations, light intensity and exposure time. Bevacizumab was showed to maintain affinity for binding to recombinant human VEGF-165 up to 15 days *in vitro* (Basuki et al., 2017).

A dual drug release system, acting by near-infrared light (808 nm) stimulation was developed and tested by Jiang et al. (2019). Nanogel-crosslinked thermosensitive hydrogels, embedded in gold nanorods (AuNR) were prepared by the precipitation polymerization of poly (*N*-isopropylacrylamide) (NIPAAm) with MBA as crosslinker and the hydrogels were prepared by radical polymerization of NIPAM with nanogels as crosslinker. Two types of drugs are encapsulated, one in the nanogel (doxorubicin) and the other in the AuNR-enriched hydrogel (curcumin). NIR light provokes a heating effect in the AuNRs, which induces a volume phase transition of the hydrogel. As a result, the drugs are released. In the first cycle, 19% of curcumin and 10% of doxorubicin were released. Curcumin is released first, and doxorubicin is released second (Jiang et al., 2019).

### Drug Administration

Non-invasive drug administration methods represent a very worthy objective but in clinical ophthalmology this is very long-term challenge. For eye treatment, these methods need to strongly improve tissue penetrability and targeting while reducing drug dispersion levels. As stated above, the structural complexity of the eye is a serious obstacle, especially when drugs have to reach the back segment. On the other side, eye barriers exclude several types of treatments, like the topically administered ones.

Anti-VEGF IVT injections, although less aggressive against drugs’ structural conformation, necessary for the effectiveness of their therapeutic principles, are not efficient. This is due to the small percentage of the drug capable of reaching the target tissue, mainly due to its dispersion and a rapid deterioration in the aqueous medium. Furthermore, IVT injections are associated with multiple complications, like infections, which can cause permanent loss of vision (Mason et al., 2008; Bhavsar et al., 2009). Consequently, multiple injections (monthly or bimonthly) are necessary to treat chronic eye diseases, which ultimately increases infection risks, patients’ discomfort and caregiving complexity.

Biomaterial-based drugs administration to extend the time between injections is the most promising approach for short-term and mid-term clinical solutions. In recent years, the use of biomaterials has been widely studied and characterized as a transport vehicle for drug administration in the eye, especially for non-biodegradable implants, colloidal systems and hydrogels. Solid carriers improve visual recovery speed, with lower vision loss risk, and avoiding multiple injections-associated side effects. However, they can increase intraocular pressure as well as to induce local adverse effects and cataract progression due to traction exerted on the vitreous humor. Solid carriers have also an inherent risk of tissue damage. As mentioned above, an ideal biomaterial for drug delivery must be able (i) to preserve physical and chemical integrity of the transporting substance (ii) to prevent its dispersion in the eye tissues/media (iii) to make it reach the target tissue and (iv) to encapsulate a large amount of drug in the smallest possible volume.

### Retention of Bioactivity Within Drug Delivery Depots

Most of the molecules employed in AMD treatment formulations are sensible to external factors such as temperature, pH, and enzymatic activity (Oo and Kalbag, 2016), and, like conventional drugs formulations, they exhibit chemical stability problems. If we focus on macromolecules, such as proteins, chemical stability challenges pairs with physical stability problems related to their tertiary structure, the maintenance of which is determines the bioactivity of the molecule (Rakesh, 2019). Chemical stability is disrupted when covalent bonds are broken and/or created in the molecule; in contrast, physical degradation occurs when non-covalent forces required to maintain the secondary, tertiary, or quaternary structure of the molecule are broken (Deb et al., 2018). Encapsulating drugs on biomaterials avoid some of these stability problems, which prevents an initial exposure of the drug to the external environment. Drug encapsulation has other benefits like the controlled drug release and decreased need for new doses administration. Moreover, the porosity of the material provides a high surface area-to-volume ratio, enhancing drug loading capacity and improving drug release profile. Nevertheless, drug encapsulation compromises bioactivity stability, with many drugs losing it when formulated in a drug delivery depot (Koerselman et al., 2020).

Large molecules are more prone to lose bioactivity via cleavage or conformational changes when encapsulated. This loss of bioactivity can be influenced by the distribution of the drug within a fibrous material (Tang, 2015). An adequate distribution of the drug far from the surface, within the material, also avoids burst release profiles (Awwad et al., 2018). Moreover, the methods used in the encapsulation process play a great role in the preservation of the bioactivity. For instance, hydrosoluble molecules are more like to maintain their bioactivity if they are exposed to less organic solvents during the process (Wilkinson et al., 2017). Another strategy that has proven to preserve drug bioactivity is to embed drugs in electrospun fibers, with hydrophilic solution core (Saraf et al., 2009). Besides, coaxial fibers fabricated by coaxial electrospinning (Qin, 2017), have shown greater potential in maintaining bioactivity and extended-release of drugs than conventional electrospun fibers (Saraf et al., 2009). Activity preservation faces other challenges in drug encapsulation in nanofibrous scaffolds. Here, drug suspension has to have good solubility, be as homogeneous as possible, be regularly immobilized in the material, and be capable to maintain its bioactivity. Needless to say, these requirements are difficult to meet, and both, pre- and post-fabrication methods need a radical improvement to reach the goals (Tiwari and Tiwari, 2013).

The principal challenges of refillable non-biodegradable implants capable for prolonged drug delivery periods [among the most advanced DDD, currently under clinical trials (Seah et al., 2020)] are to increase the volume capacity of the devices and, at the same time, to achieve a slow and uniform release speed. The idea is to dispose of large amounts of drug, to prevent the dispersion of drug molecules, as well as to slow down drug spreading and degradation rate and, consequently, to reduce interventions frequency to <1/year (Bansal et al., 2016; Rupenthal, 2017; Shen et al., 2018). Functionalize biomaterials by embedding specific drug molecules can provide a sustained, diffusion-independent drug release format (Bansal et al., 2016; Rupenthal, 2017; Shen et al., 2018), adaptable to the desired release kinetics by adjusting the degradation dynamics of the employed scaffold.

Port delivery system and micropumps have been efficient in both, preclinical and clinical trials for chronic retinopathies’ treatments (Campochiaro et al., 2011; Haghjou et al., 2011; Pirmoradi et al., 2011; Kang-Mieler et al., 2020), respectively. PDS systems are one of the very few transport systems that have been able to successfully complete clinical trials, with controlled long-term diffusion of the drug: a 220 patients Phase II clinical trial with Ranibizumab reported good PDS tolerance and maintenance of the therapeutic levels during the 9 months of the study (Campochiaro et al., 2019). However, benefits are counterbalanced by collateral risks, either related to the surgical processes or to the non-biodegradability of the implant which may lead to inflammatory processes. Indeed, in the above trial, about 5% of the patients developed surgery-originated vitreous hemorrhage.

### Technological Solutions

Hydrogels are very promising materials for sustained ophthalmic drug delivery. Their high-water content makes them very biocompatible and transparent and their porous structure acts as a protective barrier against denaturant environmental agents while allowing a large amount of drug to be loaded (Kang Derwent and Mieler, 2008; Matanović et al., 2014; Anwary et al., 2018). “Intelligent” hydrogels are able to specifically react in presence of external environmental factors like pH or temperature (Kang Derwent and Mieler, 2008; Matanović et al., 2014; Seah et al., 2020).

Several preclinical and clinical trials provided evidence for sustained drug supply, improvement of lesions and retinal functionality, with little or no toxicity of the biomaterials (Turturro et al., 2011; Agrawal et al., 2012; Drapala et al., 2014; Yu et al., 2015; OTX-IVT, 2020). However, hydrogels present several disadvantages like sterilization difficulties, risk of harm to biopharmaceuticals due to chemical crosslinking reactions, risk of toxic effects caused by polymerization initiator, presence of toxic residuals after polymerization, and difficulties in the control of drug release rate and degradation kinetics due to water absorption during swelling (Kanjickal et al., 2008; Karajanagi et al., 2011; Hammer et al., 2015; Kirchhof et al., 2015a; Baino and Kargozar, 2020).

Colloidal biofunctionalized materials have been designed to provide a format independent sustained drug release (Bansal et al., 2016; Rupenthal, 2017; Shen et al., 2018). Administration route, release rate and diffusion of the drug into the tissue directly depend on the size, structure and stability of the employed molecule (Seah et al., 2020). Drug release can also be tuned to the desired kinetics by adjusting the degradation dynamics of the scaffolding biomaterial.

Liposomes can incorporate either hydrophobic or hydrophilic compounds (e.g., anti-VEGF). They have adjustable drug release kinetics, phospholipids are metabolized as the liposome is being degraded and, compared to polymeric microparticles, they have longer half-time and lower immunogenicity and toxicity (Seah et al., 2020). Furthermore, their surface can be modified with proteins or polymers, providing additional functionalities, such as light or chemical activation, allowing specific and precise pharmacological release (Baino and Kargozar, 2020). Verteporfin was the first liposome-based drug used for photodynamic therapy of neovascular AMD, improving the topical release of bevacizumab with the use of surface-modified liposomes (Bressler et al., 2002; Fenton and Perry, 2006; Lajavardi et al., 2007). In animal models, several IVT- administered liposomes-formulated DDD have reached >1 month sustained anti-VEGF release rates (Abrishami et al., 2009; Mu et al., 2018). However, it is still unknown how liposomes interact with physiological processes, being difficult to predict the duration of sustained supply (Honda et al., 2013). For example, their stability can be compromised by the enzymatic activity or the pH of the eye, while liposomes themselves can be destabilized and ingested by macrophages (Yatvin et al., 1980; Moghimi et al., 1989). Furthermore, they have a limited drug encapsulation capability; complications with sterilization procedures and they present a risk of blurred vision after IVT injections is high (Baino and Kargozar, 2020).

Nanoparticles can encapsulate molecules, proteins, peptides and vaccines, as well as hydrophilic and hydrophobic biological macromolecules and their low cytotoxicity makes them good candidates for safe eye DDD. Their physical and chemical characteristics can be modified to fit specific pharmacological objectives (Hans and Lowman, 2002).

They have achieved several weeks of slow and sustained drug release, with some therapeutic effects, like a reduction of neovascularization damages and some improvement of the visual function (Varshochian et al., 2013, 2015; Tanetsugu et al., 2017; Narvekar et al., 2019; Qiu et al., 2019).

Despite their advantages, these systems have a low protein encapsulation efficiency (<60% for microparticles and <30% for nanoparticles), high initial release rates (20–50% of the drug in 24 h), incomplete release and loss of bioactivity of the drug during release (Yeo and Park, 2004; Manoharan and Singh, 2009; Herrero-Vanrell et al., 2014). Movement of microparticles has also been observed from the target site (Morirero et al., 1991). To limit such movement it has been proposed the simultaneous use of injectable hydrogels, to limit particles’ mobility and allow a prolonged drug release (Kang-Mieler et al., 2017). These particulate-hydrogel systems offer advantages over each of the platforms separately, as the release time is extended and the initial burst of the drug is reduced (Osswald and Kang-Mieler, 2015, 2016). Besides, since the number of microspheres suspended in the hydrogel can be controlled, the total amount of drug delivered can be controlled without changing the volume and injectability of the system (Osswald and Kang-Mieler, 2015, 2016; Bhatt et al., 2019; Liu et al., 2019c). Also, these hydrogels combined with microparticles are biocompatible and inhibit the expression of VEGF (Wang et al., 2018). However, studies to date only consist of *in vitro* and *in vivo* preclinical trials, so studies in patients have not yet been achieved. Finally, unlike other PLGA-based applications which have been proven to be safe, the small size of the micro/nanoparticles systems has, to date, limited a through test of biocompatibility and safety of the materials (Sakurai et al., 2001).

## Conclusion

To date, AMD is an untreatable neurodegenerative disease that represents a major and growing worldwide public health burden. The use of biomaterials for the sustained administration of drugs in the treatment of AMD is a promising methodology but it needs a long personal-, material- and time-effort to approximate clinical application. The immense majority of the biomaterials are still far from performing clinical trials. One of the main problems of the proposed therapies is their limited capacity for drug storage. IVT injectable and surgically implantable devices are the less elegant and oldest technology DDD, not lacking serious side effect consequences. However, they are the only available therapeutic offer for AMD patients, and they will continue to be in the near future. Improvement of these technologies is and will be extremely important for both, AMD suffering persona and for the pharma industry. Hydrogels are promising candidates due to the number of biodegradable and biocompatible components, in addition to their structural characteristics (resistance, transparency, water-holding capacity, etc.). Colloid particles are postulated as better candidates, but the development of real alternatives is still in the beginning. Probably the best therapies will combine several of the above materials and strategies to increase therapeutic effects, to reduce interventions frequency and to decrease the risks associated with such interventions.

## Author Contributions

NJ-D and AG-D: bibliographic research and information synthesis, AMD course, writing the manuscript, equal contribution. MF-A, NS-B, and NA-L: bibliographic research and information synthesis. FA-M: clinical aspects. JP-R, FR, GG, and DK: biomaterials aspects. DG-N: biomaterials, cell and tissue therapy. GG, JP-R, DK, and DG-N: manuscript revision. FP: manuscript design, information synthesis, supervision, writing the manuscript. All authors contributed to the article and approved the submitted version.

## Conflict of Interest

The authors declare that the research was conducted in the absence of any commercial or financial relationships that could be construed as a potential conflict of interest.

## Footnotes

1 ILUVIEN. *190 Micrograms Intravitreal Implant in Applicator - Summary of Product Characteristics (SmPC) - (emc)*. Available online at: https://www.medicines.org.uk/emc/medicine/27636#AUTHDATE (accessed August 15, 2020).

## Abbreviations

A2E: *N*-retinyl -*N*-retinylidene ethanolamine
AC-H: alginate-chitosan hydrogels
Ac-HA: acylated hyaluronic acid
AMD: age-related macular degeneration
ARPE-19: human retinal pigment epithelial cell line-19
AuNPs: gold nanoparticles
BRB: blood–retinal barrier
BrM: Bruch’s membrane
CPP: cell penetrating peptide
D-AMD: Dry Age-Related Macular Degeneration
DDD: drug delivery device
DEACM: 7-(diethylamino) coumarin-4-yl] methyl carboxyl
Dex-SH: thiol-functionalized polysaccharide dextran
EMA: European Medicines Agency
EPC: poly (ether ester urethane)
ESHU: poly (ethylene glycol)-poly (serinol hexamethylene urethane)
FDA: US Food and Drug Administration Agency
IAPB: International Agency for the Prevention of Blindness
IL: interleukins
IVT: intravitreally
MBA: *N*, *N* ′-methylenebisacrylamide
MEMS: microelectromechanical system
mPEG-PLGA: methoxy-poly (ethylene glycol) block-poly lactic-co-glycolic acid
mPEG-PLGA-BOX: methoxy-poly (ethylene glycol)-block-poly (lactic-co-glycolic acid)
PCL: polycaprolactone
PDMS: polydimethylsiloxane
PDS: port delivery system
PEG: poly (ethylene glycol)
PEG-DA: polyethylene glycol diacrylate
PEG-PLA: poly (ethylene oxide)-poly (D, L-lactic acid)
PEG-PLLA-DA: poly (ethylene glycol diacrylate)-co-(L-lactic acid)
PEG-PLLA-DA/NIPAAm: poly (ethylene glycol) -co- (L-lactic-acid) diacrylate/*N*-isopropylacrylamide
PEOzPCL-PEOz: Poly (2-ethyl-2-oxazoline)-b poly (ε -caprolactone)-b-poly (2-ethyl-2-oxazoline)
PGA: polyglycolic acid
PLA: polylactic acid
PLGA: poly(lactic-co-glycolic) acid
PLLA: poly (L-lactic acid)
PLLA/PLGA: poly (L-lactic acid)/poly (lactic-co-glycolic acid)
PLV: phospholipid vesicles or liposomes
PNIPAAm: Poly *N*-isopropylacrylamide
PPG: poly (propylene glycol)
PVA: poly vinyl alcohol
RPE: retinal pigment epithelium
TKI: tyrosine kinase inhibitor
TRL: clinical trials
UV: ultraviolet
VEGF: vascular endothelium growth factor
VS-HA: vinylsulfone functionalized glycosaminoglycan hyaluronic acid
HA-VS/Dex-SH: thiolated functionalized hyaluronic acid and thiolated dextran
W-AMD: Wet Age-Related Macular Degeneration
WHO: World Health Organization.
